# Reply to Correlation between pterygium and dry eye: diagnostics and risk factors not considered

**Published:** 2022

**Authors:** Leidiane Adriano, Etiene Lorriane de Souza Person, Isvander Gustavo de Souza Persona, Regina Celia Nucci Pontelli, Eduardo M. Rocha

**Affiliations:** 1 Departamento de Oftalmologia, Otorrinolaringologia e Cirurgia de Cabeça e Pescoço, Faculdade de Medicina de Ribeirão Preto, Universidade de São Paulo, Ribeirão Preto, SP, Brazil

Dear Drs. Cornejo and Levano,

Thank you for the observations on our article by Adriano et al.^(1)^, recently published in ABO.

Two major points were raised: one is related to the most accurate diagnosis of pterygium. The authors agree with your observation that a slit-lamp examination would improve the predictive value of our diagnosis. The term “presumed” was intended to alert the reader to this limitation, and the design of the study visiting the population in their homes was in part responsible for this limitation. The second point is related to more relevant risk factors to be correlated with the presence of pterygium in this population. Again, the authors agree with you about the relevance of the suggested factors; however, the intention was to explore other hypotheses, and the scope was limited by the transversal design of the study.

Once again, thank you for your interest in our study and for calling attention to these limitations.

Sincerely,

Eduardo M. Rocha

Corresponding Author

## References

[1] Adriano L, Persona EL, Persona IG, Pontelli RC, Rocha EM. (2021). Correlation between the presumed pterygium with dry eye and with
systemic and ocular risk factors. Arq Bras Oftalmol.

